# *In hospite* and *ex hospite* architecture of photosynthetic thylakoid membranes in *Symbiodinium spp.* using small-angle neutron scattering

**DOI:** 10.1107/S1600576725007332

**Published:** 2025-08-28

**Authors:** Robert W. Corkery, Christopher J. Garvey, Judith E. Houston

**Affiliations:** a Department of Material Physics, Research School of Physics Australian National University Mills Road Canberra ACT 0200 Australia; b Heinz Maier-Leibnitz Zentrum (MLZ) Technische Universität München Lichtenbergstraße 1 Garching85748 Germany; c European Spallation Source ERIC Box 176 LundSE-221 00 Sweden; NSRRC, Taiwan

**Keywords:** small-angle neutron scattering, SANS, photosynthesis, thylakoids, corals, *Symbiodinium*, *in hospite* studies

## Abstract

We demonstrate that small-angle neutron scattering (SANS) can resolve the architecture of photosynthetic thylakoid membranes in live symbiotic algal cells, both extracted from and living inside their respective hosts (*ex hospite* and *in hospite*, respectively). This enables a new non-destructive approach to probing thylakoid organization in coral symbioses, relevant to understanding the mechanisms of coral bleaching. A biologically realistic triple-vesicle model, guided by electron microscopy and established biochemical constraints, was fitted to SANS data from live *Symbiodinium* associated with both the coral analogue *Aiptasia* and the reef-building coral *Acropora*. The resulting compartment scattering length densities, together with established biochemical constraints, define a limited compositional range that supports the plausibility of the structural solution. These fits capture key scattering features and yield dimensional parameters, including inter-thylakoid (IT) gap widths, with uncertainties small enough to test models of stress-related membrane rearrangement. A focused covariance analysis shows that this SANS framework can resolve an IT-gap expansion of ∼2.4 nm with >7σ sensitivity, sufficient to distinguish structural changes proposed in thylakoid stress-response models. This provides a robust baseline for future live-cell studies.

## Abstract 2

Small-angle neutron scattering is used to detect signals from the photosynthetic membranes of symbiotic algae living inside and outside their host corals and anemones. A model is constructed for the scattering that allows the architecture of the triple membrane stack be understood in living organisms, with implications for their physiology.

## 1. Introduction

Coral reefs are ecologically significant habitats whose productivity, biodiversity and resilience depend on a photosynthetic symbiosis between dinoflagellate algae (*Symbio­dinium*) and anthazoan marine invertebrates (Bowen & Collits, 2012 ▸). Coral bleaching, in which *Symbiodinium* are lost from host tissues, can presage ecological collapse (Gilmour *et al.*, 2013 ▸). Recent models propose that thylakoid membrane reorganization in *Symbiodinium* plays a key role in stress responses and bleaching onset (Slavov *et al.*, 2016 ▸; Brown *et al.*, 1995 ▸). A detailed structural understanding of this system, particularly its *in vivo* dynamics, could therefore help predict coral health under climate-driven temperature stress (Brown *et al.*, 2022 ▸).

In single-celled organisms such as *Symbiodinium*, photosynthesis captures solar energy via protein–pigment complexes embedded in stacked thylakoid membranes within the chloroplast. This light energy drives carbon fixation, building energy-dense molecules to support algal and host metabolism. The organization of these membranes – lipid bilayers forming compartmentalized stacks – underpins the storage and conversion of chemical potential. In benthic marine ecosystems, such symbiotic photosynthesis contributes significantly to primary productivity (Larkum, 2003 ▸; Hoppenrath *et al.*, 2014 ▸). Understanding how thylakoid structure responds to environmental stress is therefore a key question in coral reef ecology. While this apparatus is tuned to ambient light, temperature and nutrient conditions, anthropogenic stressors can disrupt its function. Understanding how such perturbations alter thylakoid organization could reveal early indicators of physiological breakdown and offer insight into coral resilience or vulnerability.

Small-angle neutron scattering (SANS) provides a probe of three-dimensional organization in living cells. Because neutrons are an intrinsically non-destructive probe, SANS can track membrane rearrangements in metabolically active cells suspended in D_2_O-based media to enhance contrast (Shou *et al.*, 2020 ▸). Although scattering patterns are readily obtained, extracting unique structural insight is often limited by the low information content of data and biological heterogeneity (Pedersen *et al.*, 2014 ▸). Careful modelling is needed to resolve meaningful architectural changes.

The thylakoid membrane architecture and its physiological responses have been extensively probed in higher plants and algae using SANS (Nagy & Garab, 2020 ▸; Ünnep *et al.*, 2014 ▸; Nagy *et al.*, 2011 ▸; Posselt *et al.*, 2012 ▸; Holm, 2004 ▸; Kirkensgaard *et al.*, 2009 ▸). The dense membrane content, well defined length scales and ordered compartmentalization yield distinct features in SANS curves, which correlate closely with known ultrastructures (Jakubauskas, 2018 ▸; Jakubauskas *et al.*, 2019 ▸).

The pioneering study of Liberton *et al.* (2013 ▸) and the subsequent work of Li *et al.* (2016 ▸) on metabolically active cells demonstrated sensitivity to dynamic thylakoid re­arrange­ments. However, their analysis overlooked contributions from form factor scattering and density correlations within stacked membranes, often interpreting features solely as Bragg peaks assigned to features where the coherence length was in some cases only one unit cell. More refined models are needed to capture both the periodic structures and their internal structural motifs.

Recent work (Jakubauskas *et al.*, 2019 ▸; Jakubauskas *et al.*, 2021 ▸) showed that combining structure and form factor terms yields more realistic SANS models for thylakoid bilayers. Here we will examine a similar model, an adaptation of the lamellar stack model of Nallet *et al.* (1993 ▸) – with explicit background treatment and quantitative fitting, and constrained by real-space observations from electron microscopy – to resolve the three-compartment architecture of *Symbiodinium* thylakoid triplets.

In this way, SANS offers a route to resolve membrane rearrangements *in vivo* – under changing light or temperature – with sufficient sensitivity to detect subtle shifts in thylakoid organization. This is particularly relevant to hypotheses linking stress-induced changes in inter-thylakoid gap (IT-gap) spacing, on nanometre scales, to bleaching pathways [see *e.g.* Slavov *et al.* (2016 ▸)].

**Figure 1:**
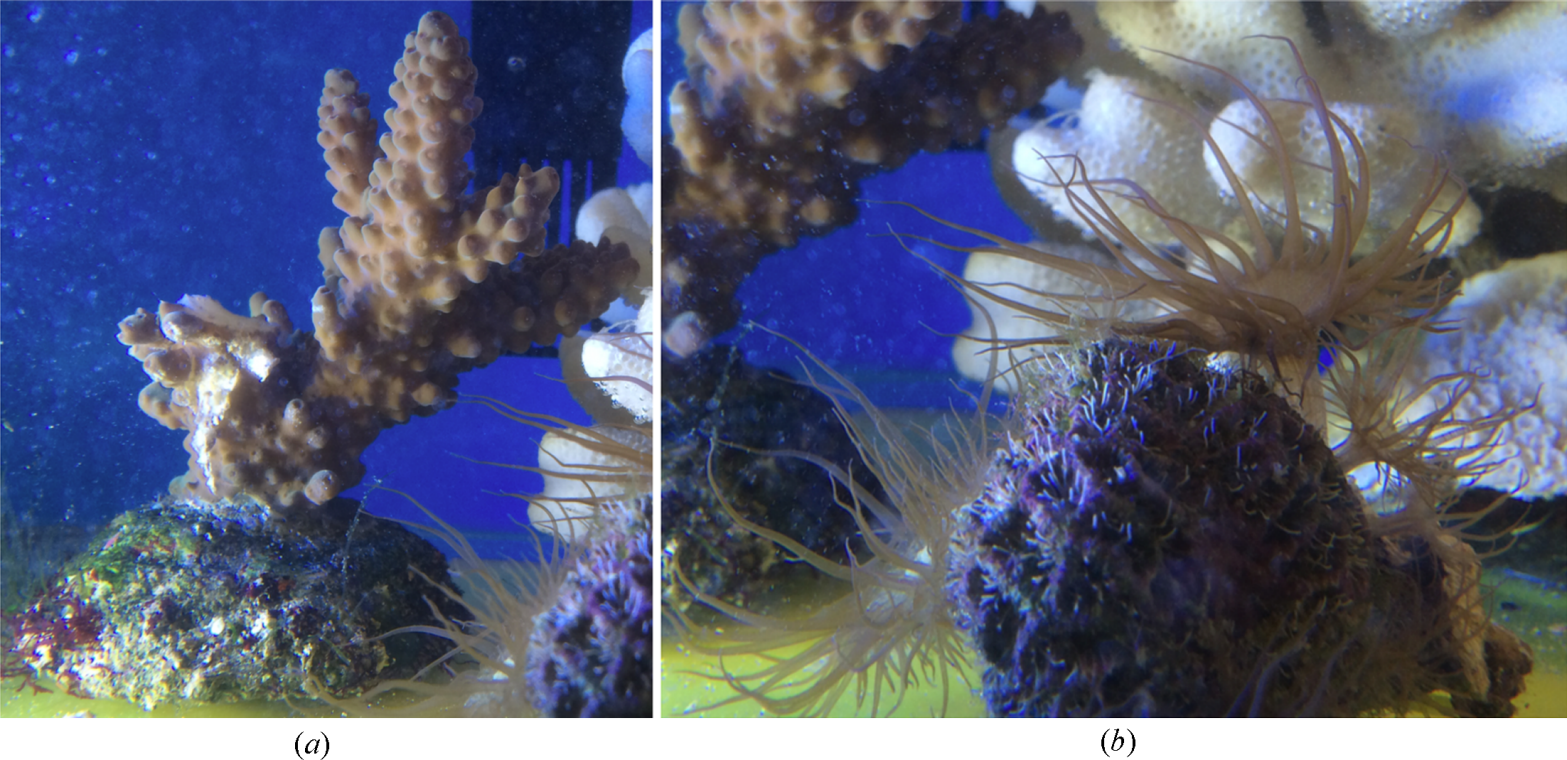
(*a*) The staghorn coral *Acropora* and (*b*) the glass anemone *Aiptasia* used in the SANS experiments here. The round base of the staghorn coral is approximately 5 cm diameter. The same base is seen on the left-hand side of panel (*b*).

Two symbionts have been examined in this study: the common reef-forming staghorn coral *Acropora* and the glass anemone *Aiptasia*. Both organisms are shown in Fig. 1 ▸. *Aiptasia* is a model organism for studying the symbiotic relationship between algae and corals (Dunn *et al.*, 2002 ▸). The brown colour in the coral and anemone is due to chloro­phyll and perdinin pigmentation (Niedzwiedzki *et al.*, 2014 ▸; van Amerongen & Croce, 2013 ▸) within the respective endosymbiotic dinoflagellate algal (*Symbiodinium*) cells held within the tissue of each. Live algal cells were either freshly extracted from the respective host coral or anemones for SANS measurements or directly measured inside the living anemone.

## 2. Methods and materials

### 2.1. Preparation and handling of organisms

Samples were prepared for scattering using deuterated salt water, the salt being a commercially available sea salt (Red Sea Europe, Verneuil, France) used for growing corals in home aquaria. *Acropora*^1^ and *Aiptasia* specimens were purchased from a commercial coral supplier for home aquaria; the original geographical sources are unknown, although all *Aiptasia* specimens were obtained together and thus likely represent the same genetic stock. The healthy specimen of *Acropora sp.* was approximately 10–12 cm in length with multiple small branches. The multiple *Aiptasia sp.* specimens were anchored on several small rocks, with individuals varying from less than 1 mm up to 5–7 cm diameter in their fully extended state.

The coral and anemone samples were kept for several weeks in ideal growth conditions in a small reef tank located at Newcastle University, UK. *Symbiodinium* algal cells were extracted from the coral by breaking a small branch or branches from the main mass and fragmenting it with a mortar and pestle. In the initial extraction on day zero, the relatively dense and adherent *Symbiodinium* cells were purified using multiple cycles of differential settling by relatively gentle centrifugation, isolation of the dark-brown algal cell layer and re-suspension in fresh sea water at approximately 25°C. A similar procedure was used to isolate the algal cells from an *Aiptasia* individual. This preparation method closely follows that described by Domart-Coulon *et al.* (2011 ▸), which preserves cell integrity and viability for at least 96 h post-isolation. These cells were then transported overnight, under carefully controlled conditions to ensure viability, from Newcastle, UK, to Garching, Germany. The following day (day 1) the extracted cells were centrifuged further and the light sea water replaced with heavy sea water of the same salinity (approximately 34.7–35.0 p.p.t.). After several further centrifugation cycles (6000*g* for 5 min, three times) in heavy (deuterated) sea water (at 34.6–34.9 p.p.t. salinity) the samples were ready for SANS measurements.

*Symbiodinium* living inside an individual *Aiptasia* specimen (approximately 5 cm fully open diameter with approximately 0.5 mm diameter tentacles) were prepared for *in vivo*/*in hospite* measurement on day 2 by carefully removing the base of the anemone near its attachment point and then exchanging light sea water for heavy sea water of equal salinity and temperature several times over an hour. The substitution of heavy sea water induced a radial shrinkage of the tentacles by a factor of about 5–10× in length compared with the same anemone in light water, leading to much higher local density of the symbiotic cells in the former. This gave the deuterated *Aiptasia* an almost black appearance and a new size of approximately 5–7 mm diameter. The *Aiptasia* specimen maintained healthy turgor for several hours in D_2_O, indicative of vitality during the experiments reported here. Samples were not dark-adapted and light intensity varied only according to laboratory conditions. Measurements were run in local low white lighting conditions available within the beam hall, with the temperature controlled to approximately ±0.5°C at 20 and 32°C. SANS measurements of *Symbiodinium* could be made in the host organism, *in hospite*, or extracted, *ex hospite*.

### 2.2. SANS experiments and raw data treatment

SANS was performed on the KWS-2 instrument at the Heinz Maier-Leibnitz Zentrum (Garching, Germany; Radulescu *et al.*, 2015 ▸). In both cases samples were cylindrical quartz cuvettes (Helma Gmbh, Müllheim, Germany). The empty quartz cell was employed for background subtraction; this background was used rather than the cell containing the D_2_O-based buffer as we found that any quantitative estimation of the contribution of the latter required a precise understanding of the relative volume of living cells in the sample interrogated by the neutron beam. All measurements were made in 1 mm thick quartz cells which accommodated a 10 mm diameter neutron beam.

A continuous SANS curve of absolutely scaled intensity, *I*(*q*) [*q* = (4π/λ)sin(θ/2), where λ is the wavelength and θ the scattering angle] was made by combining three instrumental configurations which collected the isotropic scattering patterns. All data acquired on the KWS2 instrument used an incident neutron wavelength of 5 Å and detector distances of 2, 8 and 20 m with collimations of 8, 8 and 20 m, respectively, to yield a *q* range of 0.0038 to 0.49 Å^−1^. The scattered intensity was collected on a ^3^He detector with an active area equivalent to 0.9 m^2^. Each raw scattering data set was corrected for detector sensitivity, electronics background and sample transmission and converted to scattering cross-section data using the instrument software *QtiKWS* (https://www.qtisas.com). These data were converted to the absolute scale (cm^−1^) through reference to the scattering from a secondary standard sample (Plexiglas).

### 2.3. Fast Fourier transform (FFT) image analysis

**Figure 2:**
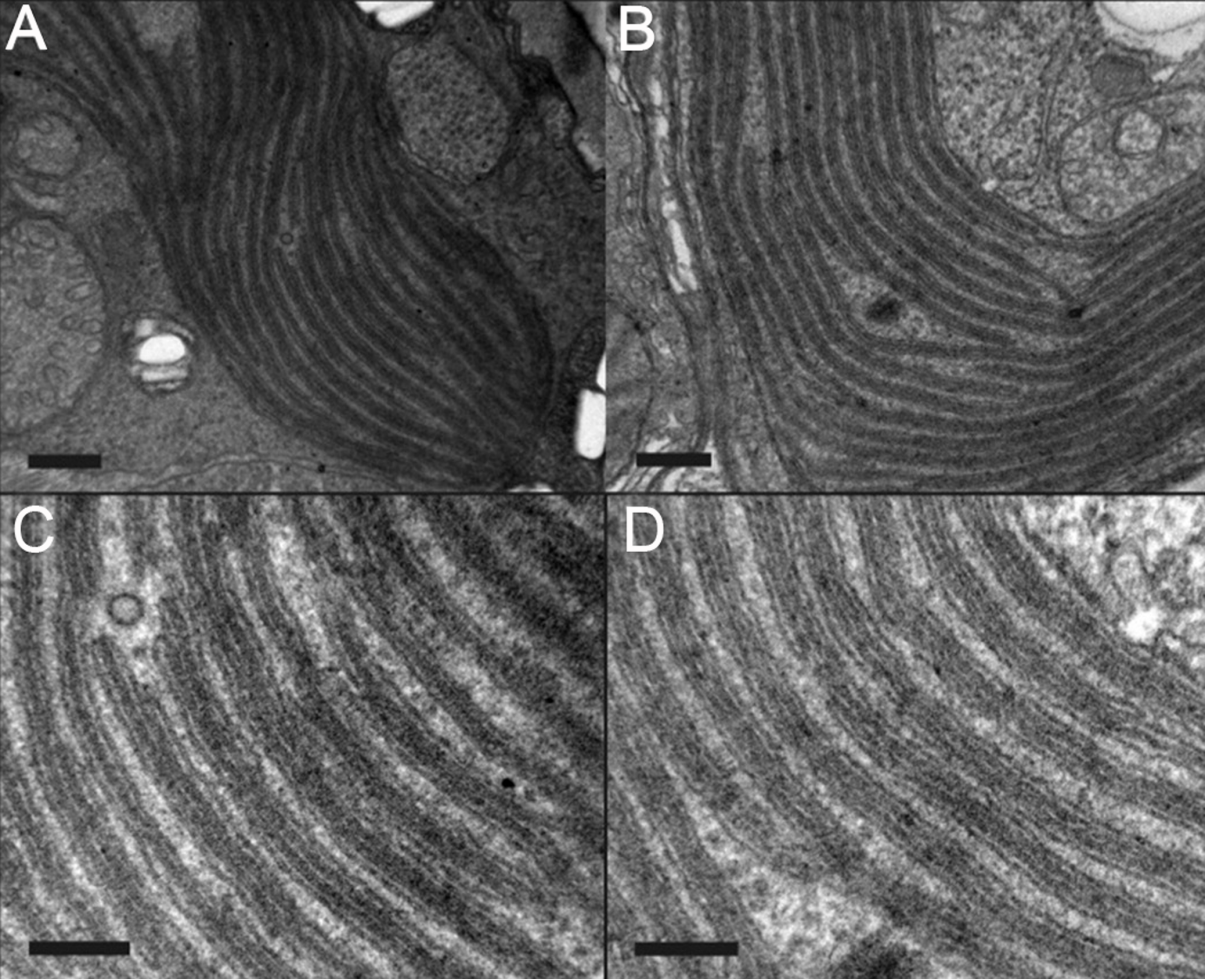
Transmission electron micrographs of thylakoid stacks inside *Symbio­dinium* cells. Reproduced from Fig. 2 of Slavov *et al.* (2016) ▸, copyright (2016), with permission from Elsevier. Membrane contrast was enhanced with OsO_4_ and uranyl acetate treatments. Panels (*a*) and (*c*) represent cells adapted to the dark at 24°C. Panels (*b*) and (*d*) represent cells incubated at 31°C with 600 µmol photons m^−2^ s^−1^. Scale bars represent 200 nm in panels (*a*) and (*b*) and 100 nm in panels (*c*) and (*d*). Thylakoids are typically stacked into triplets and these triplets form the repeating units seen in these images.

**Figure 3:**
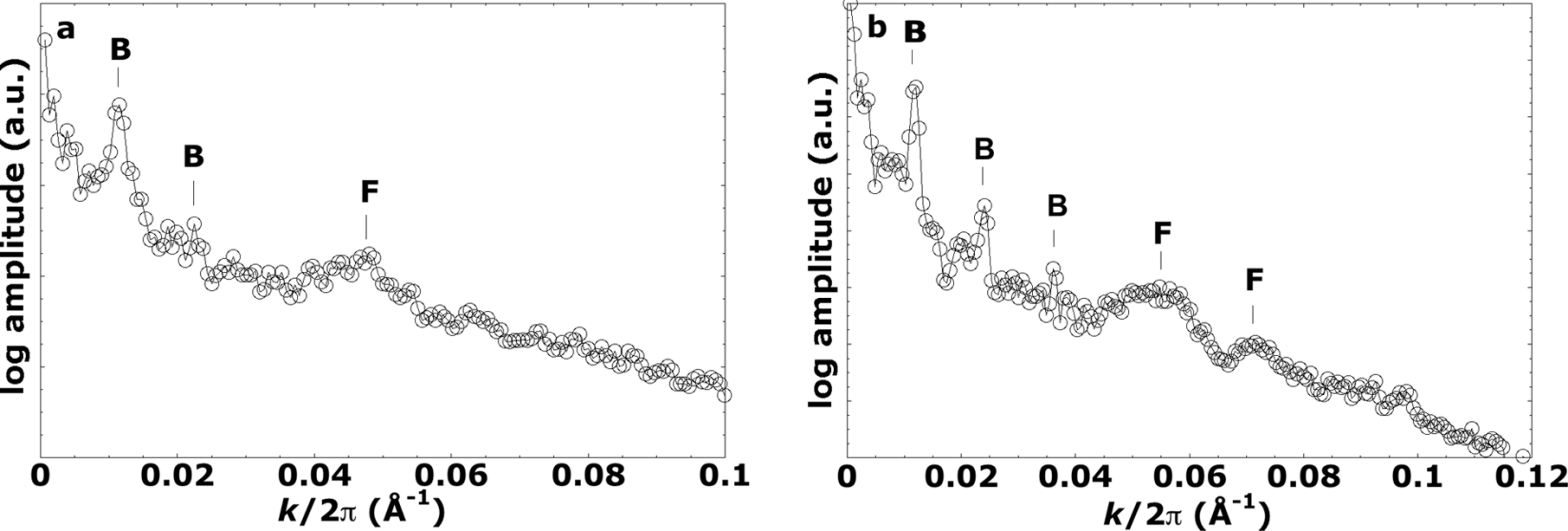
Radially averaged FFTs of TEM images of thylakoids in *Symbio­dinium* (Slavov *et al.*, 2016 ▸). (*a*) 24°C and dark-adapted, (*b*) 31°C and light-irradiated. The magnitude of the **k** vector (inverse distance) on the *x* axis of each power spectrum has been scaled to equivalent *q* (= *k*/2π) for ease of comparison with the SANS spectra obtained here. Clear maxima labelled B are assigned to the first- and higher-order repeat distances (*RD*) of thylakoid triplets. The respective *RD* values are (*a*) 556 ± 82 Å and (*b*) 537 ± 40 Å. The estimated uncertainty of the *RD*s is taken from the half-width at half-height on the lower-*q* side of the respective first-order peak. These maxima are consistent with the thylakoid *RD*s measured directly from the image. The peaks labelled F are 2D image equivalents of form factor peaks in a 3D SANS experiment. These correspond to the sub-structure inside the 2D projection of repeating triplets in the real-space images. They arise from unknown grey-level image contrast functions, in this case set by selective interaction of the thylakoid components with the OsO_4_ and uranyl acetate employed by Slavov *et al.* (2016 ▸) in enhancing their image contrast. Since the contrast functions are not known, we do not use these any further here.

Two-dimensional (2D) FFTs of TEM images were generated with the FFT Javascript function within *ImageJ* (Version 1.51s; Rueden *et al.*, 2017 ▸), which employs an FFT algorithm to generate the FFT log-amplitude spectrum. Two-dimensional FFTs were derived from images Fig. 2(*e*) and 2(*f*) of Slavov *et al.* (2016 ▸) for *Symbiodinium* at 24 and 31°C, the former dark-adapted and the latter incubated under irradiating light [Figs. 2 ▸(*a*) and 2 ▸(*b*), respectively]. Using the ‘radial profile angle’ plugin in *ImageJ*, a radial average was made of a narrow sector of each 2D FFT with an angular sweep of approximately 20° centred on an azimuth perpendicular to the repeating pattern to obtain 1D power spectra. Each radially averaged FFT was scaled from the original wavevector **k** to obtain equivalent *q* values on their respective *x* axes for easier reference to the SANS data sets (see Fig. 3 ▸).

## 3. Results and discussion

**Figure 4:**
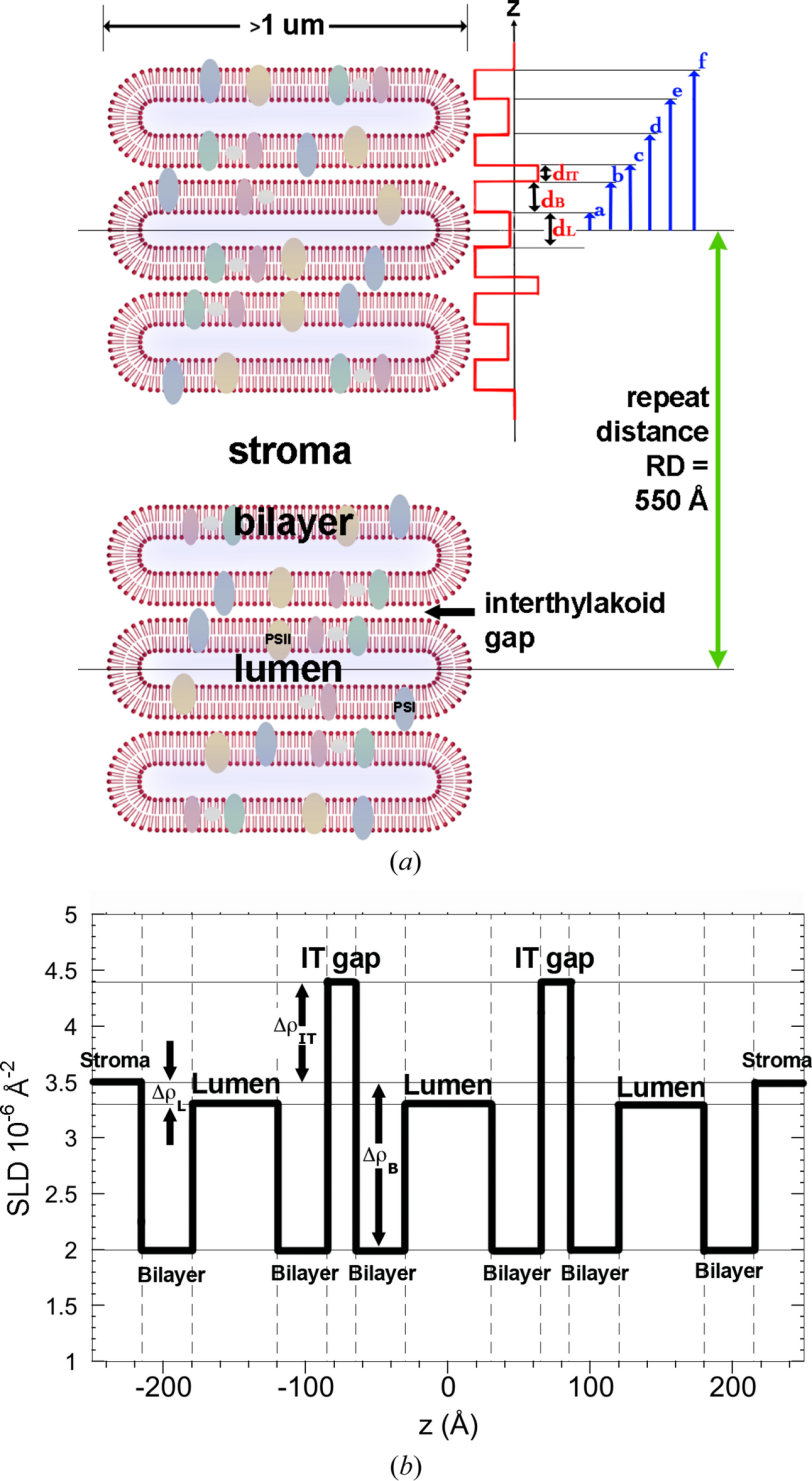
The triple-vesicle model for photosynthetic dinoflagellate thylakoid structures. Here Δρ_IT_, Δρ_B_, Δρ_L_ and *d*_IT_, *d*_B_, *d*_L_ are the SLD contrasts and thicknesses of the IT gap, bilayer and lumen, respectively. These are used in calculating the form factor of the triple-vesicle stack. Adjacent stacks are separated by a distance *RD*, which is used in the calculation of the structure factor. See the supporting information for details of the calculation of the form factor *P*(*q*) shown in equation (3). Coloured ovals in the lipid bilayers represent membrane proteins.

The form factor for thylakoid triplets in the coral symbiont *Symbiodinium* is based on the typical triple-stacked structure of thylakoid membranes in chloroplasts of dinoflagellates (Schnepf & Elbrächter, 1999 ▸; Slavov *et al.*, 2016 ▸; see Fig. 2 ▸). Each thylakoid comprises a membrane surrounding a lumenal space with the various photosynthetic biomolecules embedded in or associated with the membrane. Here we assume each thylakoid is a flattened vesicle, stacked into triplets of individual vesicles and separated by an inter-thylakoid space that is contiguous with the stroma of the chloroplast, with a characteristic repeat distance (*RD*) between stacks of thylakoid triplets. For simplicity the model is further simplified by taking a 1D section through the series of stacks, noting the various thicknesses and assigning a stepped scattering length density (SLD) function to represent the average composition of each component, namely the lipid membrane, lumen and IT gaps. The model is represented in Fig. 4 ▸. Fig. 4 ▸(*a*) shows the 2D model with an incidental line section, and Fig. 4 ▸(*b*) shows the corresponding SLDs and thicknesses on the *z* axis, representing this section in expanded form.

To model scattered intensities due to membranes, we take the approach used by Nallet *et al.* (1993 ▸) to estimate the scattering from lyotropic liquid crystal lamellar phases, except here we model a sextuplet of membranes (representing three thylakoids) as the basic scattering unit instead of a singlet (Nallet *et al.*, 1993 ▸), or a doublet in the case of Jakubauskas *et al.* (2019 ▸). The expression used here for intensity is given in equation (1),
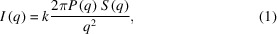
where *P*(*q*) is the form factor, *S*(*q*) is the structure factor and the factor *k* represents the effective scaling of the intensity due to the number of scattering units.

Nallet *et al.* (1993 ▸) accounted for fluctuations of individual layers about a mean position with a Gaussian distribution about that mean by introduction of a Caille parameter into the structure factor – see expression (A1) in the *Appendix* (supporting information). A high Caille value represents a rigid membrane and a low value a flexible membrane. By varying the Caille parameter from zero to one, the higher orders of the Bragg reflections range from undamped to fully damped, with non-zero values essentially representing polydispersity in the spacing between thylakoid stacks in the present case.

We first repeat the work of Jakubauskas and co-workers (Jakubauskas, 2018 ▸; Jakubauskas *et al.*, 2019 ▸; Jakubauskas *et al.*, 2021 ▸) to model scattering from cyanobacteria with a stack of single thylakoids using the same form factor expression as they used in equation (1) above. The single-vesicle model form factor is
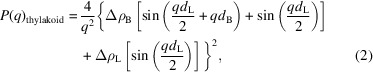
where the symbols in equation (2) are the same as those in Fig. 4 ▸. Note that in this single-vesicle model the inter-thylakoid space is not a component of the form factor since it comprises only the bilayer surrounding a single lumen.

Figs. A9(*a*) and A9(*b*) in the supporting information show that we have successfully implemented the same model and obtained precisely the same fit using their data and parameter values (Table A4) for the cyanobacteria *Synechocystis* PC 6803 (Jakubauskas *et al.*, 2019 ▸). In implementing the calculation of *P*(*q*) in our triple-vesicle model, we introduced a lumenal polydispersity term σ_L_ on the basis of observed variations in the lumen thickness observed in TEM images obtained by Slavov *et al.* (2016 ▸). Essentially, one calculates *I*(*q*) for multiple different models, varying the lumen thickness in each case with a Gaussian distribution around a mean and a characteristic standard deviation. A Gaussian distribution was chosen as a relatively simple distribution of the lumenal polydispersity. A weighted sum of these models then gives the final *I*(*q*), including the variation in the lumen widths. In the triple-vesicle model, lumenal polydispersity is implemented by having the same Gaussian distribution of lumen widths occurring in all three lumens of a single stack. This is essentially simplifying the lumenal width variance to be the same locally and differing only between separate triple stacks, and keeps the number of fitting parameters associated with defining the form factor lower than would otherwise be the case.

Having achieved the same fit for a single model as Jakubauskas *et al.* (2019 ▸) gave us confidence in testing the triple-vesicle model with the form factor given by
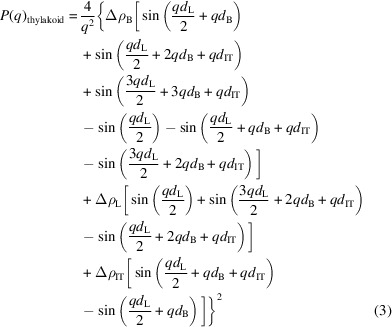
Equation (3) was generated using the 1D section shown in Figs. 4 ▸(*a*) and 4 ▸(*b*), and combined with the structure factor of equation (A1) into equation (1) to yield *I*(*q*) for the final triple-vesicle model. Calculations of *I*(*q*) were coded and run as a formula script in *Kaleidagraph* (Version 4.5, Synergy Software, Reading, Pennsylvania, USA). Running *Kaleidagraph* formula scripts allowed graphical plotting of each new model in real time, providing immediate visual feedback after a parameter change and thus aiding a relatively quick refinement via visual inspection.

### 3.1. Refinement and parameter sensitivity

For all samples, initial parameters were refined visually to obtain reasonable starting values. For the extracted *Aiptasia* (20°C) sample, this visual fit seeded the χ^2^ minimization used to obtain final parameters and formal uncertainties. For the other two samples (extracted from *Acropora* and *in hospiteAiptasia*), visual fits were used and uncertainties are qualitative. For visual refinement we have chosen to fix the Δρ_B_ value, corresponding to the lipidic part of the membrane, to a relative value of −1.45 × 10^−6^ Å^−2^ in agreement with an estimate for higher plants by Jakubauskas *et al.* (2019 ▸). We set an initial value for the bilayer thickness to 36 Å. This value is as expected for the lipid core of a bilayer in higher plants and algae and is mid-range for various SANS studies in general (Jakubauskas *et al.*, 2021 ▸). An initial *RD* value is that derived from TEM images (Figs. 2 ▸ and 3 ▸ and Table 1 ▸). Next, the lumen and IT-gap widths were refined, their respective values also estimated from the TEM images. Without any hard restrictions we found that, in refining the model parameters via manual pattern matching with the experimental SANS curves, Δρ_IT_ and Δρ_L_ were manually refined to values less than |1 × 10^−6^| Å^−2^, in accordance with the expected relatively low contrast with the surrounding stroma. The IT-gap SLD being closer to that of protein than that of D_2_O suggests a higher protein fraction than might be expected for a stromal-contiguous aqueous gap. Thus the fitting of Δρ_IT_ and *d*_lumen_ values was remarkably intolerant to much variation once physically reasonable values for *RD*, *d*_bilayer_, Δρ_bilayer_ and Δρ_IT_ had been fixed so that the positions of the main modelled peaks were coincident with those of the observed data sets. Smaller features like the slopes at the edge of the large experimental peaks or small peaks could then be used to tune the fit of the model of lumenal polydispersity. Finally, all parameters were manually refined in finer and finer increments until no better fit was found. This resulted in departures from all of the initial estimates based on TEM images or previous studies, other than the SLD of the bilayer which was held absolutely fixed.

For the extracted *Aiptasia* (20°C) sample, formal uncertainties are given in Table 2 ▸ derived from χ^2^ analysis. For the two visual-fit samples, the stated tolerances are qualitative estimates based on the observed sensitivity of the fits (see supporting information Section A3 for a description of this fitting procedure). In future studies, full χ^2^ analysis of all samples will allow stronger comparison across biological conditions and experimental states.

In some cases, the structure sampled during a SANS measurement may represent a mixture of biological states. In this case a mix of models may be warranted, requiring additional information as the number of unknowns rises. One of these will be discussed below as a possible end member that may mix into other samples (see discussion of Fig. 6).

Despite its geometric simplifications, the triple-vesicle model reproduces the key scattering features across all samples. These robust cross-validated parameters provide the basis for assessing stress-induced thylakoid rearrangements, such as IT-gap expansion (Slavov *et al.*, 2016 ▸) during bleaching events.

**Figure 5:**
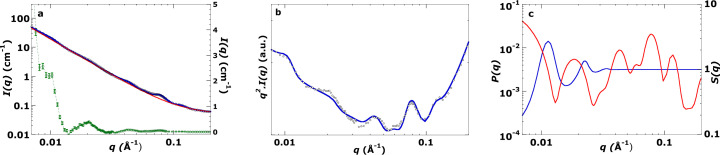
(*a*) Log–log SANS plot of *Symbiodinium* algae extracted from coral analogue *Aiptasia* (the linear scale on the right-hand side belongs to the green curve – see below). Measurement at 20°C in 100% D_2_O. Small black circles are SANS data points. The blue curve is the log–log fit refined using the χ^2^ minimization method (see Section A4). χ^2^ = 4.31 (*q* = 0.008–0.2 Å^−1^). The red curve is the background function refined using the χ^2^ minimization method. The green curve is a linear plot after subtracting the background function (red curve) from the SANS data (scale on right-hand side). Error bars on the green curve are the 1σ uncertainty in intensity (*dI*) at each *q*. The SANS signal is well above the uncertainty in the green curve. (*b*) For every (*q*, *I*) pair in panel (*a*), the intensity *I* is multiplied by *q*^2^ to obtain *q*^2^*I*(*q*) versus *q* curves. This plot style aids in visualizing the maxima and minima from low to high *q*, and provides a direct comparison of the fitted curve after χ^2^ minimization. The close agreement between χ^2^ refined and visual fits for this sample provides confidence in applying visual fits for the other cases. Model parameters were first refined by visual iteration (Section A3); these values seeded the χ^2^ minimization described here. (*c*) Log–log plots of model components *P*(*q*) and *S*(*q*) used in the construction of the fit curve for *Symbiodinium* extracted from *Aiptasia* shown in panel (*a*). The red curve is the model form factor *P*(*q*) curve calculated using equation (3) with the parameters given in Table 2, and the blue curve is the model *S*(*q*) curve calculated using equation (A1) with the parameters given in Table 2.

**Figure 6:**
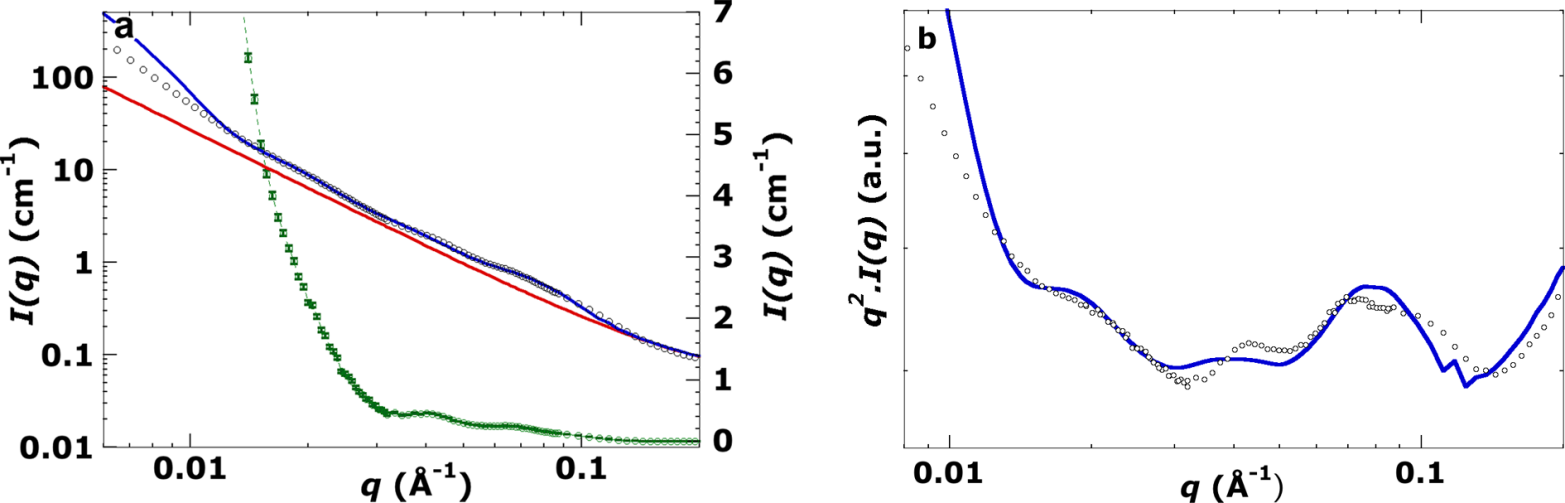
(*a*) Log–log SANS plot of live *Symbiodnium* algae extracted from the live coral *Acropora*. Measurement at 20°C in 100% D_2_O. Small black circles are SANS data points. The blue curve is the fit refined using the visual inspection method (see Section A4 of the supporting information for more details of this method). χ^2^ = 8.2 (*q* = 0.012–0.2 Å^−1^). The red curve is the background function refined by the visual inspection method (Section A3). The green curve is the linear plot obtained after subtracting the background function (red curve) from the SANS data (scale on right-hand side). Error bars on the green curve are the 1σ uncertainty in intensity (*dI*) at each *q*. Note that the signals are well above the uncertainty in the green curve and that no peak is seen near *q* = 0.01 Å^−1^ (see text for further discussion). (*b*) The same data (black circles) and fit (blue line) plotted as *q*^2^*I*(*q*) versus *q*.

**Figure 7:**
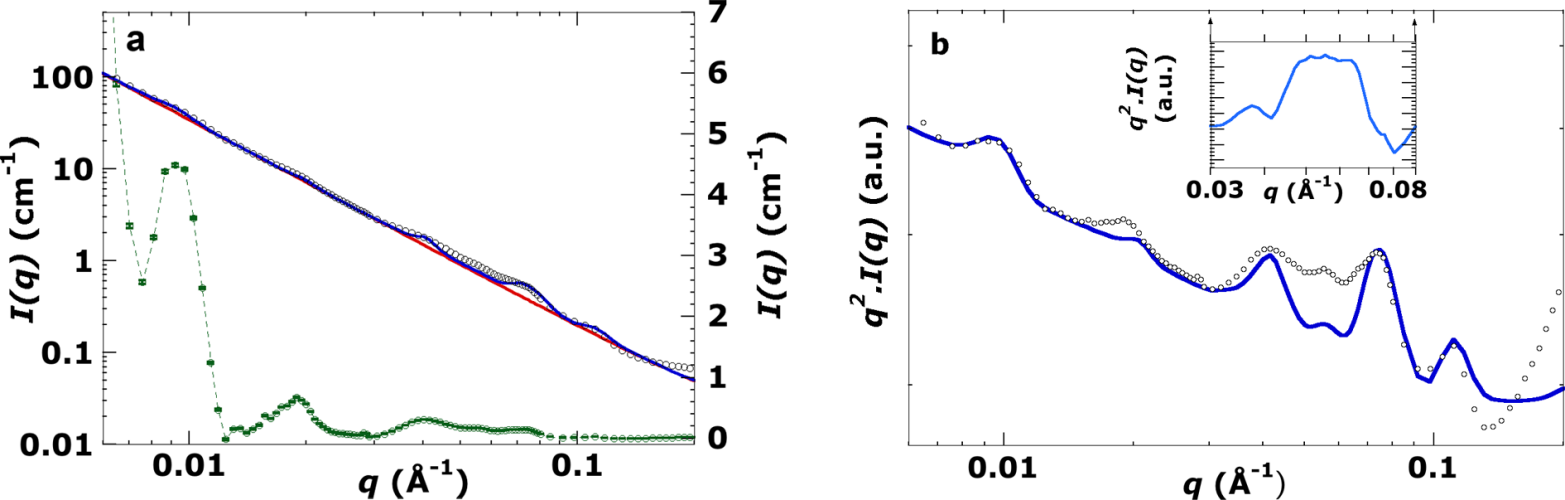
(*a*) Log–log SANS plot of live *Symbiodnium* algae in a live coral analogue, the glass anemone *Aiptasia*. Measurement at 32°C in 100% D_2_O. Small black circles are SANS data points. The blue curve is the fit refined using the visual inspection method (see Section A4 of the supporting information for more details of this method). χ^2^ = 22.8 (*q* = 0.008–0.15 Å^−1^, excluding *q* = 0.04–0.07 Å^−1^ – see text). The red curve is the background function refined by the visual inspection method (see Section A3 of the supporting information). The green curve is the linear plot obtained after subtracting the background function (red curve) from the SANS data (scale on right-hand side). Error bars on the green curve are the 1σ uncertainty in intensity (*dI*) at each *q*. Signals are well above the uncertainty in the green curve. (*b*) The same data as plotted in panel (*a*), now plotted as *q*^2^*I*(*q*) versus *q*.

Fig. 5 ▸ shows the observed data and the final fitted SANS model from live *Symbiodinium* algae extracted from *Aiptasia*. Because the *Acropora* extract lacked a detectable thylakoid stacking peak (see Fig. 6 ▸ and discussion) and the *in hospite* sample included additional host-derived scattering (see Fig. 7 ▸ and discussion), formal χ^2^ minimization was not applied to these cases. As this is a demonstration study, a single well resolved χ^2^ analysis sufficed to validate the model. The χ^2^ minimization fitting parameters are listed in Table 2 ▸. The strong peak near *q* = 0.01 Å^−1^ corresponds to the first-order Bragg peak (*RD* = 555 ± 22 Å), consistent with the 556 Å peak in the FFT of the TEM image (Fig. 3 ▸, Table 1 ▸). The fit also matches higher-*q* features and the minor peak near *q* ≃ 0.06 Å^−1^, which is sensitive to lumenal polydispersity. The good match between χ^2^-based and visual fits for this sample (Table 2 ▸) provides confidence in applying visual fits to the other two cases.

For a visual fit of the SANS data for *Symbiodinium* extracted from *Acropora* (Fig. 6 ▸), our triple model was set to one layer, equivalent to setting *S*(*q*) = 1, so that the effective scattering is from isolated unstacked triple thylakoids. The absence of any significant scattering above the intrinsic linear decay of the log–log plot near *q* = 0.01 Å^−1^, or of any sharp point in the broad maximum centred near *q* = 0.02 Å^−1^, is as expected for unstacked or highly disordered stacks of thylakoid triplets. Inspection of the TEM images in Fig. 2 ▸ shows that stacked triplets can ‘delaminate’ from other triplets. Thus we attempted to fit the data for this sample to a single triplet. The main features are reasonably fitted, namely the maxima near *q* = 0.04 Å^−1^ and *q* = 0.08 Å^−1^. Repeat experiments not shown here on distinct extracts from the same *Acropora* fragment reveal the same basic pattern – a lack of any primary Bragg peak. *Aiptasia* extracts always had a low-*q* peak indicative of stacks of triples. This suggests that the low-*q* peak was either masked (perhaps from scattering due to adherent calcium carbonate skeletal particles that were not removed during extraction by crushing/centrifugation) or lacked ordered stacking. In either case the visual fitting here required a very high lumenal polydispersity and a lower average lumenal width. *RD* and the Caille parameter have no meaning when no triplet stacking is involved. Nevertheless this fitting of essentially only the form factor may be useful for fitting destacked thylakoid triplets. The process of triplet destacking may be a precursor to loss of membrane appression, noted by Slavov *et al.* (2016 ▸) to occur in dinoflagellates that are sensitive to temperature-induced bleaching.

The SANS curve shown in Fig. 7 ▸(*a*) is scattering from *Symbiodinium* cells located inside the host anemone (*in hospite*). Compare this with the SANS curve of *Symbiodinium* cells extracted from the host (*ex hospite*) shown in Fig. 5 ▸(*a*). A notable difference between these two cases (*in* and *ex hospite*) is the significant extra scattering intensity shown in the small inset [Fig. 7 ▸(*b*)] after subtracting the fit from the experimental curve. In repeated extracts from *Aiptasia*, we never observed this contribution to the scattering, but we always observed the same feature when the *Symbiodinium* was in the symbiotic partner tissue, *i.e. in hospite*. This points to the likelihood that the scattered intensity in this region arises from the host tissue, likely a collagenous hydrogel (Singer, 1974 ▸), rather than from the thylakoids of its *Symbiodinium* guest. It is beyond the scope of this study to analyse this. This region was excluded from the χ^2^ calculation of the visual fit. For the remainder of the pattern the fit is good in the regions of scattering above background and the fitted values are close to those seen in Fig. 5 ▸, but *RD* and the thylakoid thickness are now larger than those obtained by image analysis (Table 1 ▸). The main Bragg peak is well resolved, so the stacking repeat distance is well estimated *in hospite* and in extracted or *ex hospite* cells.

For the extracted *Aiptasia* (20°C) sample, the visual-fit parameters seeded the χ^2^ minimization described here. For the other two samples, visual fits were used. Although the χ^2^ analysis used here is based on local one-parameter-at-a-time sensitivity scans (varying each parameter through its χ^2^ minimum with others held constant), this still provides useful insight into the degree to which the model is constrained. It is not a full multi-parameter covariance analysis and does not capture all possible parameter interactions. However, in this system, many parameters are either tightly linked to biological priors (bilayer thickness, SLD contrasts, number of layers) or influence only specific regions of the fit (*e.g.RD*, Caille). The χ^2^ curves (Figs. A1–A4) show how steeply χ^2^ increases when moving away from the best-fit point and, together with inspection of fit sensitivity plots (Figs. A5–A8), provide an effective means of assessing parameter sensitivity and coupling.

### 3.2. Covariance refinement confirms IT-gap thickness resolvability

To assess whether our model can distinguish stress-linked structural changes, we tested its sensitivity to the IT-gap thickness (*d*_IT_) and its corresponding contrast (Δρ_IT_). A 3 × 3 χ^2^ grid spanning *d*_IT_ = [14.9, 16.6, 18.3] Å and Δρ_IT_ = [6.3, 9.2, 12.1] × 10^−7^  Å^−2^ produced a best fit at 16.6 Å, with 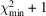 spanning ±1.7 Å, identical to the one-parameter uncertainty (see Table 3 ▸). In contrast, a high-stress grid centred on 40 Å yielded χ^2^ values more than 30 (χ^2^) units above the threshold across all points. The two states form non-overlapping confidence bands. This confirms that the model can resolve an IT-gap expansion of ∼24 Å with >7σ sensitivity, enough to detect changes consistent with stress-induced membrane reorganization.

Having established that our model can resolve a physio­logically relevant IT-gap expansion through both single-parameter and two-parameter tests, we now consider the broader fit structure. Despite the nominal 14 parameters, the real freedom of the model is strongly limited by biochemical and structural constraints. The modest additional uncertainty from the compositional corridor should formally be added in quadrature to the χ^2^-derived errors; however, this remains small relative to the shifts of interest. The fitted compartment SLDs and volumes must remain physically realistic, summing to 100% and matching known membrane and lumen properties, which forces the solution into a narrow compositional window. For this reason, even though only one formal χ^2^ sensitivity analysis was performed, and based on local scans, the combination of structural constraints and the compositional envelope shown in Table A3 strongly support the plausibility of the global solution. Because the IT-gap thickness is tightly determined by the SANS fit, while its composition is constrained by independent biochemical limits, the dimensional conclusions drawn here, such as testing models of IT-gap expansion, are robust to potential circularity. The apparent local nature of the 1D scans does not undermine this (plausibility that the solution is global), as alternative solutions outside this compositional space would violate basic biological constraints.

### 3.3. Resolving stress-linked membrane shifts

The covariance analysis of *d*_IT_ and its paired contrast parameter (Δρ_IT_) showed that the stress-linked gap expansions fall well outside the 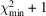 confidence region, while the low-stress state remains tightly bounded. This non-overlap confirms that the model resolves a ~24 Å gap shift with >7σ sensitivity, sufficient to distinguish the structural change proposed in stress-response models (*e.g.* Slavov *et al.*, 2016 ▸). Although only the low-stress configuration was observed here, this result demonstrates that SANS, when paired with compositionally constrained modelling, can detect physiologically meaningful membrane reorganization. This establishes a quantitative threshold for detecting stress-linked thylakoid remodelling *in vivo*.

### 3.4. Compositional envelope from SANS

On the basis of the fitted SLDs and known biochemical constraints, Table A3 defines the broadest biologically realistic volume percent windows for each chloroplast compartment (stroma, IT gap, lipid bilayer and lumen) that simultaneously satisfy the three measured contrasts (IT gap 0.90 ± 0.05 × 10^−6^ Å^−2^, bilayer −1.45 ± 0.05 × 10^−6^ Å^−2^, lumen −0.15 ± 0.05 × 10^−6^ Å^−2^). Because the SLD is defined by linear mixture rules, the constraints are likewise linear: (i) all fractions sum to 100%, (ii) components occupy only their proper niches (*e.g.* head groups in aqueous zones, tails in the core) and (iii) each compartment’s contrast remains within its experimental band. An iterative ‘push-to-boundary’ approach in this convex space locates the true outer bounds of permissible compositions. The resulting SLD corridor (±0.08 × 10^−6^ Å^−2^) narrows the absolute-baseline freedom to a single likely solution – one that explicitly includes chlorophyll, peridinin and plastoquinone in the membrane core. While not formally unique, this compositional envelope provides a robust baseline for interpreting changes in thylakoid stacking, such as stress-induced IT-gap expansion, in future live-cell SANS studies.

Together, the χ^2^ refined fit and compositional envelope demonstrate that live-cell SANS of *Symbiodinium* now achieves spatial resolution on the scale of ∼10–20 Å. This is sufficient to resolve the magnitude of IT-gap expansion (∼20 Å) implicated in Photosystem I reorganization under thermal stress (Slavov *et al.*, 2016 ▸), supporting the use of SANS to test key models of bleaching-related thylakoid dynamics. With this baseline established, future studies can extend these insights across varied physiological states and environmental conditions.

### 3.5. Biological significance and future prospects

SANS proves to be a significant and practical tool for assessing thylakoid spacing in live organisms, particularly where membrane stacking reflects the photosynthetic state. Its statistical advantage is clear: millions of cells (∼10^7^ to 10^8^ per run; see Section A6) are measured simultaneously, in contrast to TEM where only small altered populations are imaged. This study demonstrates that SANS can reproducibly resolve thylakoid spacings *in vivo* and, together with the validated triple-vesicle model and derived compositional corridor (Table A3), provide a robust baseline for interpreting structural shifts. These findings now offer a sound basis for SANS-based studies aimed at directly testing models of thylakoid reorganization during stress events such as coral bleaching. Future experiments, such as tracking temperature responses or clade-specific effects, are now well motivated.

## 4. Conclusions

This study demonstrates that small-angle neutron scattering can detect and resolve thylakoid membrane structures within live *Symbiodinium* cells, both *ex hospite* and *in hospite*. Using a biologically realistic triple-vesicle model, refined by χ^2^ minimization and guided by established biochemical constraints, we have achieved fits that robustly reproduce all key scattering features. The resulting dimensional parameters, such as inter-thylakoid gap and bilayer spacings, are determined with uncertainties that are small relative to the ∼10–20 Å shifts implied in stress-induced IT-gap expansion models (*e.g.* Slavov *et al.*, 2016 ▸).

The corresponding compartment SLDs, when combined with biochemical priors, define a narrow compositional corridor that provides additional confidence in the structural solution. Although this adds modestly to the total uncertainty budget, it does not affect our ability to resolve the key structural shifts of interest. A targeted covariance analysis confirms that the model resolves an IT-gap expansion of ∼24 Å with >7σ sensitivity, defining a quantitative threshold for detecting stress-linked membrane reorganization.

This is the first rigorous demonstration that live-cell SANS can yield both structural and compositional insights into photosynthetic membranes of dinoflagellates. The validated triple-vesicle model offers a practical framework for future studies of thylakoid organization under environmental stress. We anticipate that this approach will enable quantitative tracking of bleaching-related thylakoid rearrangements *in vivo*, with potential to inform mechanistic models of coral bleaching.

## 5. Related literature

For further literature related to the supporting information, see Dodge (1969 ▸), Jacrot (1976 ▸), Kitmitto *et al.* (1997 ▸), Kurreck *et al.* (2000 ▸), Lin *et al.* (2024 ▸), Press *et al.* (2007 ▸), Sears (1992 ▸), Sharma *et al.* (2024 ▸) and Trench & Blank (1987 ▸).

## Supplementary Material

Additional information, tables and figures. DOI: 10.1107/S1600576725007332/ju5087sup1.pdf

## Figures and Tables

**Table 1:** Measurements of key distances from the transmission electron micrographs of Slavov *et al.* (2016 ▸)

Sample	*RD*† (Å)	Triple-stack thickness‡
Dark adapted, 24°C	556 ± 82	390 ± 6
*Symbiodinium* in light, 31°C	537 ± 40	410 ± 3

† Our estimates from FFTs of the images in panels (*a*) and (*b*) of Fig. 2.

‡ From direct-space measurements of the thickness of the dark triple stacks in Figs. 2(*c*) and 2(*d*). Subtracting the triple-stack thickness from *RD* gives the thickness of the stromal gap between triple stacks [white layers in Figs. 2(*a*)–2(*d*)].

**Table 2:** Refined model parameters

	Refinement method			
	*χ^2^* minimization	Visual inspection		
	*Symbiodinium Aiptasia ex hospite*, 20°C	*Symbiodinium Aiptasia ex hospite*, 20°C	*Symbiodinim Acropora ex hospite*, 20°C	*Symbiodinium Aiptasia in hospite*, 32°C
Fit parameters				
*RD* (Å)	555 ± 22	570 ± 15	–	610 ± 15
*N*_layers_	11.7 ± 19.6	9 ± 2	1	10
η_Caille_	0.26 ± 0.17	0.12 ± 0.02	–	0.05
*d*_bilayer_ (Å)	40.7 ± 1.5	40.0 ± 1	37 ± 1	36 ± 1
*d*_lumen_ (Å)	57.0 ± 2.9	57 ± 2	40 ± 5	65 ± 2
σ_lumen_ (Å)	11.1 ± 2.8	10 ± 1	35 ± 5	10 ± 1
*d*_IT_ (Å)	16.6 ± 1.7	16 ± 1	33 ± 2	27 ± 2
Δρ_B_ (×10^−6^ Å^−2^)	−1.45 ± 0.78	−1.45†	−1.45†	−1.45†
Δρ_L_ (×10^−7^ Å^−2^)	−1.47 ± 1.0	−1.8 ± 2	−7 ± 2	−1.4 ± 1
Δρ_IT_ (×10^−7^ Å^−2^)	9.2 ± 2.9	9 ± 2	0.5 ± 0.2	4.5 ± 1.0
*k*‡ (×10^6^)	1.624 ± 0.218	1.75 ± 0.02	15 ± 1	3.0 ± 0.2
*C*§ (×10^4^)	2.130 ± 0.033	2.12 ± 0.02	17 ± 2	10.3 ± 0.02
*n* §	−2.4755 ± 0.0042	−2.465 ± 0.005	−2.10 ± 0.07	−2.26 ± 0.01
*B*§ (×10^−2^)	4.66 ± 0.22	4.7 ± 0.2	4.5 ± 0.2	0.8 ± 0.2
χ^2^ fit	4.31	13.4	8.21	22.8

Derived parameters				
Stack thickness (Å)	449 ± 21	443 ± 14	408 ± 25	465 ± 16
Interstack space (Å)	107 ± 21	127 ± 14	–	150 ± 16

† The bilayer SLD contrast was fixed in the visually refined fits, so no uncertainties are included.

‡ *k* is the intensity scalar.

§ Background parameters where *I*(*q*)_bkgd_ = *B* + *Cq*^*n*^.

**Table 3:** Covariance-corrected *d*_IT_ uncertainties

Condition	Best-fit *d*_IT_ (Å)	Covariance ±1σ (Å)
Low-stress	16.62	±1.70
High-stress†	–	Rejected ( 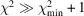 )

† All high-stress grid points lie well above 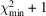.

## Footnotes

1 Species identities were not determined; formally these would be expressed as *Acropora sp*.,* Aiptasia sp.*/*spp.* and *Symbiodinium sp.*/*spp.*, but genus names alone are used throughout for readability.
