# Supplementary material for: In hospite and ex hospite architecture of photosynthetic thylakoid membranes in Symbiodinium spp. using small-angle neutron scattering
Source: J Appl Crystallogr. 2025 Aug 28;58(Pt 5):1516–25. doi: 10.1107/S1600576725007332 (PMC12502874; doi:10.1107/S1600576725007332)
Supplement: Supplementary file 1 [file j-58-01516-sup1.pdf]

JOURNAL OF
APPLIED
CRYSTALLOGRAPHY

Volume 58 (2025)

Supporting information for article:

In hospite and *ex hospite* architecture of photosynthetic thylakoid membranes in *Symbiodinium spp.* using small-angle neutron scattering

Robert W. Corkery, Christopher J. Garvey and Judith E. Houston

Appendix

Contents

A1 Structure factor expression

A2. Form factor expression

A3. Method to fit model curves to SANS data using visual inspection

A4 Method to fit model curves to SANS data using chi-squared (χ^2) minimization and uncertainty analysis

A5 Compositional envelope from SANS

A6 Estimated cell concentration in each experiment

A7. Single vesicle model and comparison with previously published work

A8. Covariance Analysis for d_{IT} versus $\Delta\rho_{IT}$

A1. Structure factor expression

Structure factor expression used here (after Nallet *et al.* 1993):

$$S(q_z) = 1 + 2\sum_{n=1}^{N-1}\left(1-\frac{n}{N}\right)\cos\left(\frac{q_z dn}{1+2\Delta q^2 d^2 \alpha(n)}\right) \times e^{-\frac{2q_z^2 d^2\alpha(n) + \Delta q^2 d^2 n^2}{2\left(1+2\Delta q^2 d^2 \alpha(n)\right)}} \times \frac{1}{\sqrt{1+2\Delta q^2 d^2 \alpha(n)}} \quad \text{(A1)}$$

Here

$S(q_z)$ is the structure factor as a function of the scattering vector, q

N is the number repeating units in stack

Δq is the instrument resolution (only applied to structure factor)

d is the spacing between repeating units

$\alpha(n)$ is the correlation function, where for small n

$$\alpha(n) = \frac{\langle(u_n - u_0)^2\rangle}{2d^2} = \frac{\eta n^2 d^2}{8}$$

or for *n* >>1

$$\alpha(n) = \frac{\langle(u_n - u_0)^2\rangle}{2d^2} = \frac{\eta}{2\pi^2}[\ln(\pi n + \gamma]d^2$$

where

u_n is the displacement in a direction normal to the membrane stack due to Gaussian fluctuations of the nth triple vesicle stack around its equilibrium position, *n.d*,

and where,

$$\eta = \frac{q_0^2 k_B T}{8\pi}\sqrt{K\bar{B}}$$

where

k_B is the Boltzmann constant

T is the temperature

K and $\bar{B}$ are the elastic constants of the triple vesicle stack

A2. Form factor expression

The form factor for theoretical scattering can be constructed from Figure 3 of the main text, noting the following relationships:

$$a = \frac{d_L}{2}$$

$$b = \frac{d_L}{2} + d_B$$

$$c = \frac{d_L}{2} + d_B + d_{IT}$$

$$d = \frac{d_L}{2} + 2d_B + d_{IT}$$

$$e = \frac{3d_L}{2} + 2d_B + d_{IT}$$

$$f = \frac{3d_L}{2} + 3d_B + d_{IT}$$

From this, the following form factor expressions for the bilayer, lumen and inter-thylakoid gap were derived:

$$F(q)_{bilayer} = \frac{2}{q}\Delta\rho_B \left[\sin\left(\frac{qd_L}{2} + qd_B\right) + \sin\left(\frac{qd_L}{2} + 2qd_B + qd_{IT}\right) + \sin\left(\frac{3qd_L}{2} + 3qd_B + qd_{IT}\right) - \sin\left(\frac{qd_L}{2}\right) - \sin\left(\frac{qd_L}{2} + qd_B + qd_{IT}\right) - \sin\left(\frac{3qd_L}{2} + 2qd_B + qd_{IT}\right)\right]$$

$$F(q)_{lumen} = \frac{2}{q}\Delta\rho_L \left[\sin\left(\frac{qd_L}{2}\right) + \sin\left(\frac{3qd_L}{2} + 2qd_B + qd_{IT}\right) - \sin\left(\frac{qd_L}{2} + 2qd_B + qd_{IT}\right)\right]$$

$$F(q)_{IT\ gap} = \frac{2}{q}\Delta\rho_{IT} \left[\sin\left(\frac{qd_L}{2} + qd_B + qd_{IT}\right) - \sin\left(\frac{qd_L}{2} + qd_B\right)\right]$$

Combining each of the contributions from the bilayer, lumen and cytosolic gap form factor expressions we find the form factor *P(q)* for the entire thylakoid stack:

$$P(q)_{thylakoid} = \frac{4}{q^2}\left\{\Delta\rho_B \left[\sin\left(\frac{qd_L}{2} + qd_B\right) + \sin\left(\frac{qd_L}{2} + 2qd_B + qd_{IT}\right)\right.\right.$$

$$+ \sin\left(\frac{3qd_L}{2} + 3qd_B + qd_{IT}\right)$$

$$\left. - \sin\left(\frac{qd_L}{2}\right) - \sin\left(\frac{qd_L}{2} + qd_B + qd_{IT}\right) - \sin\left(\frac{3qd_L}{2} + 2qd_B + qd_{IT}\right)\right]$$

$$+ \Delta\rho_L \left[\sin\left(\frac{qd_L}{2}\right) + \sin\left(\frac{3qd_L}{2} + 2qd_B + qd_{IT}\right)\right.$$

$$\left. - \sin\left(\frac{qd_L}{2} + 2qd_B + qd_{IT}\right)\right]$$

$$\left.+ \Delta\rho_{IT} \left[\sin\left(\frac{qd_L}{2} + qd_B + qd_{IT}\right) - \sin\left(\frac{qd_L}{2} + qd_B\right)\right]\right\}^2$$

A3. Method to fit model curves to SANS data using visual inspection

Manual visual fitting was used to refine initial parameters for all samples. For the extracted *Aiptasia* (20 °C) sample, this visual fit seeded the χ^2 minimisation used to obtain final parameters and uncertainties reported in the main text.. Initial model fitting was performed by visual inspection using log-log and $q^2 \cdot I(q)$ versus q plots of the SANS data. The background slope and vertical offset were first adjusted to establish the local power-law behaviour at low and high q. In parallel, FFT analysis of TEM images was used to identify the first Bragg peak and search for any higher-order peaks (e.g., only a weak second-order peak was evident in the SANS plot for *Symbiodinium* extracted from *Aiptasia* and measured at 20 °C). Real-space TEM measurements provided constraints on the lumen thickness, IT gap thickness, and approximate number of layers. Based on the limited Bragg order observed, the Caille parameter and number of layers were set accordingly. The bilayer SLD contrast was then

explored systematically, followed by refinement of all SLD values by iterative comparison to experimental curves, with particular sensitivity gained by using $q^2 \cdot I(q)$ plots to assess fit quality. The lumen polydispersity was adjusted to match the observed form factor peak widths. This manual fitting procedure produced parameter values very close to those later obtained by formal χ^2 minimisation (using 1D χ^2 vs parameter scans). Notably, for two of the three data sets reported, the manual fits alone were used, as they provided reasonable solutions (relatively low χ^2 values). The ability to judge fit quality by visual inspection in $q^2 \cdot I(q)$ space proved remarkably effective, and was used as the start point for further refinement using χ^2 analysis.

Refining the triple vesicle model – visual refinement and χ^2 minimisation

In a typical refinement, the background is the first element fit, and is largely well defined by two linear features in the log-log plot: at low q, a power law with slope ≈ 2, attributed to surface scattering from cellular membranes (Jakubauskas et al., 2019); and at high q, a constant intensity from incoherent scattering due to residual ^{1}H. Various background forms were tested; a damped power-law function $I_b(q) = B + Cq^{-n}$ was found most effective, with fewer parameters and good performance across the q-range of interest. Because the high-q background is clear, and the available q-range sufficiently broad, this component can be confidently subtracted before further fitting.

The volume of stacked thylakoid triplets that scattered is not known, even if the cell density in the beam was approximately known. This unknown scattering volume is captured in the intensity scaling term, k.

Each manual fitting was performed iteratively. Our current code allowed only a single calculation with fixed parameters without automatic refinement. Feedback was through visual pattern matching and setting new parameters and running until a visual match was not

improved further. Results are given in Table 2. While this method of pure inspection-based minimisation is informative about parameter sensitivity, it was laborious, subjective and uncertainties are not formally determined. To address this, we applied χ^2 minimisation to the *Aiptasia*-extracted dataset (20 °C), using the visual fit as a starting point.

A4. Method to fit model curves to SANS data using chi-squared (χ^2) minimization and uncertainty analysis

As described in the main text, SANS data were fitted first using a method of iterative visual inspection and parameter stepping. These visually refined models were then further refined by χ^2 analysis.

.

To estimate the χ^2 refined fits and uncertainty in model parameters we performed a local analysis based on the variation of chi-squared (χ^2) with a single parameter while holding all others fixed.

Method Overview:

1. We began with the close-fit model from visual fitting, optimized to match small-angle neutron scattering (SANS) data.

2. The parameter of interest (e.g., *RD*) was varied over a suitable range around the best-fit visual value of the model fitted to the data

3. For each parameter value, the χ^2 components were computed at each q_i value using:

$$\chi^2(q_i) = [\,(\log_{10}(I(q_i)_{exp}) - \log_{10}(I(q_i)_{model})) \,/\, (dI_i / (I(q_i)_{exp} \ln(10)\,]^2$$

where:

- $I(q_i)_{exp}$ is the observed experimental intensity

- $I(q_i)_{model}$ is the model prediction

- dI_i is the 1σ uncertainty at each point in the raw data file from the SANS instrument

The individual $\chi^2(q_i)$ components are added to obtain χ^2.

4. The resulting χ^2 values as a function of *RD* formed a well-behaved parabola in the vicinity of the minimum.

5. A quadratic fit of the form:

$\chi^2(RD) = a \cdot RD^2 + b \cdot RD + c$

was applied to the locally parabolic region.

6. The minimum was computed analytically as:

$RD_{\chi^2 \mathrm{min}} = -b / (2a)$

7. The 1σ uncertainty bounds were obtained by solving:

$\chi^2(RD) = \chi^2_{min} + 1$

which yields two *RD* values bounding the 68% confidence interval under Gaussian error assumptions.

This method follows standard practice in Gaussian error theory. See: Press *et al.* (2007).

Table A1 Parabola coefficients (of the form $y = ax^2 + bx + c$)

parameter	a	b	c
**RD**	0.0020	-2.22	628.6
**Lumen SLD**	9.44E+13	2.78E+07	14.67
**Incoh bkgd**	1.99E+05	-1.93E+04	476.1
**Bkgd scalar**	9.44E+10	-4.02E+07	4290
**Bkgd power**	5.87E+04	2.91E+05	3.60E+05
**intensity scalar**	2.11E-11	-6.86E-05	61.12
**Caille**	35.3	-18.5	7.15
**N Layers**	0.00261	-0.0611	5.12
**Lumen sigma**	0.126	-2.81	20.27
**Bilayer thick**	0.432	-35.2	720.3
**Lumen thick**	0.115	-13.1	379.2
**IT gap thick**	0.336	-11.2	97.16
**IT gap SLD**	1.21E+13	-2.24E+07	14.64
**Bilayer SLD**	1.65E+14	4.79E+08	350.4

NB. Here y is the chi-squared (χ^2) value obtained from the modelling, holding all parameters fixed but one, and then running the triple vesicle model code manually once for each parameter variable change.

Table A2 Chi-squared (χ^2) minima and uncertainties analytically from parabolas

parameter	x-value at χ^2_{min}	positive root	1σ	% uncertainty
**RD**	555.15	577.52	22.37	4.0296
**Lumen SLD**	-1.47E-07	-4.44E-08	1.03E-07	69.853
**Incoh bkgd**	0.048646	0.05089	0.0022434	4.6118
**bkgd scalar**	0.00021301	0.00021626	3.25E-06	1.5279
**Bkgd power**	-2.4755	-2.4713	0.0040975	0.16552
**intensity scalar**	1.62E+06	1.84E+06	2.18E+05	13.405
**Caille**	0.26225	0.43052	0.16827	64.162
**N Layers**	11.687	31.245	19.558	167.35
**Lumen sigma**	11.13	13.943	2.8133	25.276
**Bilayer thick**	40.699	42.22	1.521	3.7372
**Lumen thick**	57.046	59.993	2.9466	5.1654
**IT gap thick**	16.62	18.344	1.724	10.373
**IT gap SLD**	9.24E-07	1.21E-06	2.87E-07	31.114
**Bilayer SLD**	-1.45E-06	-1.37E-06	7.78E-08	5.367

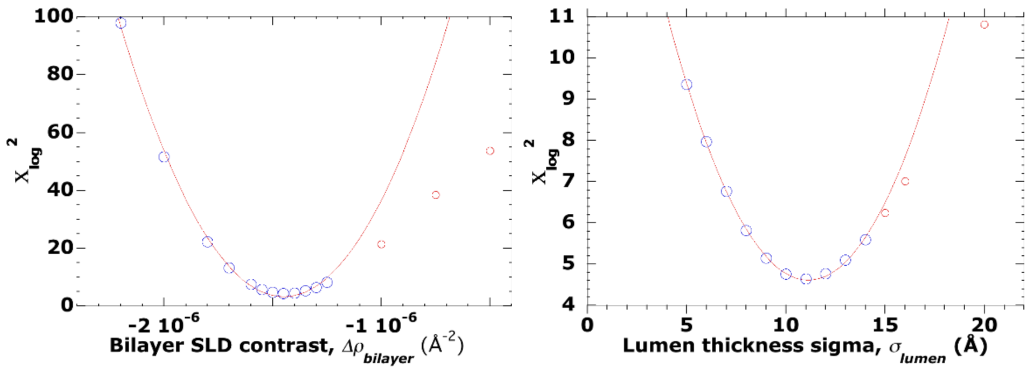

Figure A1 and Figure A2

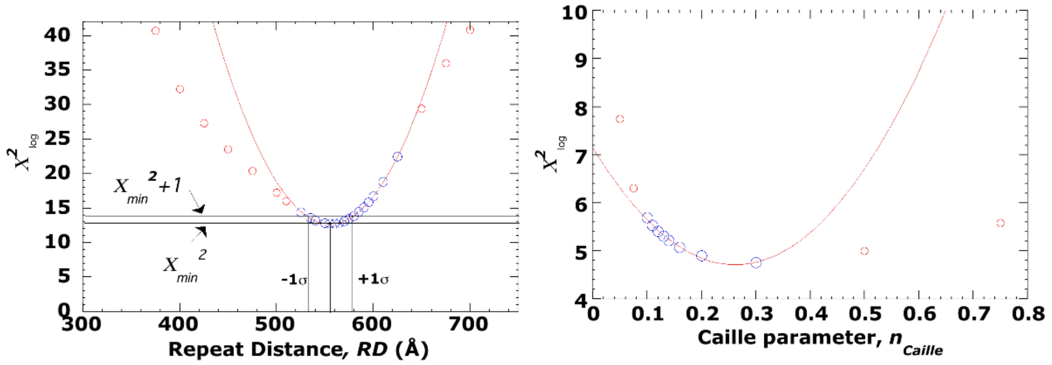

Figure A3 and Figure A4

Figures A1–A4 show representative χ^2 versus parameter curves ("χ^2 parabolas") for key parameters, illustrating how local sensitivity was assessed; Supplementary Figures A5–A8 show corresponding fit sensitivity plots, illustrating how variation in each parameter propagates through $q^2 \cdot I(q)$ space and affects the quality of the fit. χ^2 versus parameter curves for four representative model parameters: (A) Bilayer SLD, (B) Lumen sigma, (C) *RD*, and (D) Caille parameter. These illustrate the range of parameter sensitivities observed in the model: steep, well-defined minima for strongly coupled parameters (A, B), an intermediate behaviour with minor shoulder for *RD* (C), and a broad, weakly coupled curve for Caille (D), which has little effect on the overall fit.

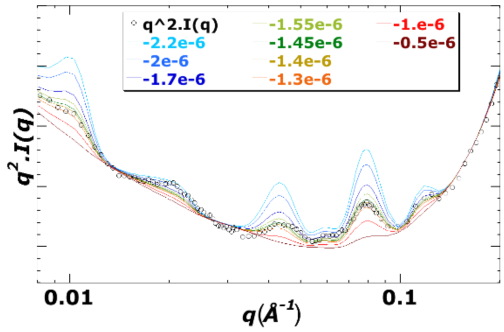

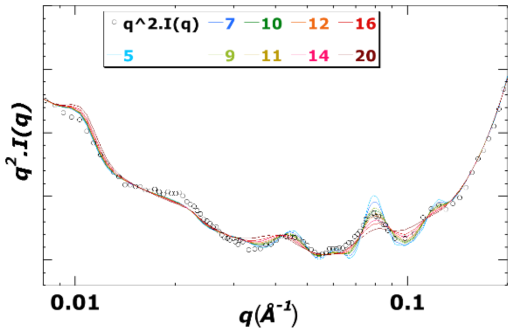

Figure A5 and Figure A6

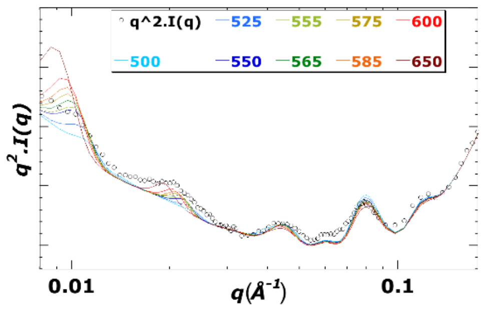

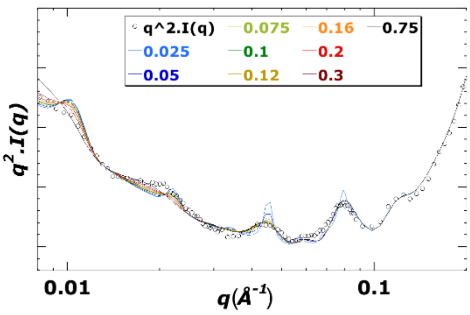

Figure A7 and Figure A8

Figures A5-A8 (*S*ensitivity or parameter coupling plots) Effect of parameter variation on $q^2 \cdot I(q)$ fits for four representative parameters: (A) Bilayer SLD (strongly coupled), (B) Lumen sigma (strongly coupled), (C) *RD* (affecting low-*q* Bragg region *q*=0.01-0.02 Å-1), and (D) Number of Layers (weakly coupled, minor fit impact). These plots illustrate how parameter variation propagates through the fit.

A5. Compositional envelope from SANS

Table A3 Composition windows satisfying fitted contrasts

Compartment	Compositional ranges (vol %) + protein exchange	Absolute SLD ($\times10^{-6}$ Å^{-2})	Δ SLD vs. stroma ($\times10^{-6}$ Å^{-2})
**Stroma**	D_2O 40–50 %; Residual H_2O pockets 10-15 %; Soluble nascent glucan 5–8 %; Neutral lipid droplets 8–10 %; Protein 8–14 % (80–90 % D); Nanostarch/ β-glucan ≤ 3 %	3.47 – 3.52	0
**Inter-thylakoid gap (16 Å)**	D_2O 50–56 %; Lipid headsgroups 22–26 %; Protein loops 20–26 % (80–90 % D)	4.37 – 4.40	0.88 – 0.93
**Bilayer core**	Water 0–5 %; Protein 50–55 % (65–80 % D); Unsaturated acyl chains 9–17 %; Chlorophyll 15–20 %; Plastoquinone 8–12 %	1.90 – 2.00	–1.55 – –1.45
**Lumen (57 Å total)**	Headgroup slabs: 17–22 % Lipid headgroups + 7–10 % protein loops (80–90 % D); Aqueous cavity: D_2O 38–46 %; Residual H_2O 18–28 %; Soluble protein 10–13 % (75–85 % D); Salts/metabolites 4–6 %	3.28 – 3.38	–0.19 – –0.14

To set the widest credible composition windows we treated each compartment—the chloroplast stroma (reference phase), the inter-thylakoid gap, the lipid bilayer, and the lumen—as a mixture of only the components that can actually occupy it (water, D-exchanged protein, lipid headgroups or tails, chlorophyll, plastoquinone, salts, carbohydrates and metabolites). Scattering-length density (SLD) is a linear sum of component volumes, so the constraints are flat: (i) volumes add to 100 %; (ii) a component appears only where it belongs (e.g. headgroups outside, lipid tails inside); (iii) the contrasts of the three internal compartments — IT-gap, bilayer, lumen — relative to the stroma must stay within the measured bands (+0.90 ± 0.05, –1.45 ± 0.05, –0.15 ± 0.05 × 10^{-6} Å^{-2}, respectively). Because both the stroma and the lumen are necessarily water-rich, their SLDs sit near the upper edge of the allowed corridor, while the bilayer core (water-poor) and the gap (headgroup and protein rich) pull lower or higher accordingly. In this flat, convex space an iterative "push each component until a boundary is touched" method reaches the same limiting faces that a formal optimiser would, so the ranges reported in Table A3 are indeed the broadest compatible with both biochemistry and the experimental contrasts. Note Table A3 lists the exact SLDs used: water, exchange-adjusted protein, MGDG/DGDG/SQDG head-groups, chlorophyll/peridinin, plastoquinone, lipid droplets and starch or its glucan precursors. Component volume-% windows in Table A3 are independent bounds; for any chosen set of values within those ranges, the component percentages are normalized so their sum equals 100 %, ensuring each compartment remains physically meaningful.

SLDs of components (and suitable concentrations within the relevant compartment of the thylakoids) were estimated from the following sources: Proteins – Jacrot (1976); algal lipids - Jakubauskas, D. (2018); Chlorophyll and peridinin content of lipid bilayers - van Amerongen and Croce (2013) and Niedzwiedzki *et al.* (2014); Plastoquinone content of lipid bilayers: Kurreck et al (2000); Glucan/starch manufacture location on stromal side of algal thylakoids – Zeeman et al. (2024) and starch in dinoflagellates - Dodge (1969). All calculations of SLDs when not otherwise available were done following: Sears (1992).

A6. Estimated cell concentration in each experiment

A rough, order-of-magnitude concentration can be obtained as follows. Electron-microscopy measurements put a *Symbiodinium* cell diameter near 10 µm (Trench & Blank, 1987), giving each cell a volume of $\approx 5 \times 10^{-16}$ m³. If the extracted pellet in a cuvette behaves like a loose random-close-packed assembly (≈50–55 % of the volume actually occupied by cells), then one millilitre (10^{-6} m³) of slurry contains about $(0.5 \times 10^{-6}\ \mathrm{m}^3)/(5 \times 10^{-16}\ \mathrm{m}^3) \approx 10^9$ cells. Allowing for the ±7 % spread in measured diameters and the ±5 % uncertainty in packing, the estimate is $(1.0 \pm 0.2) \times 10^9$ cells mL^{-1}. This figure is intended only as a practical 'ball-park' value for beam-path calculations rather than a definitive concentration. In the neutron beam path for our experiments (1 cm² area × 1 mm length = 0.10 mL), this corresponds to roughly 1×10^8 cells if the sample holder was fully filled; however with 40–70 % occupancy attained here ~$4–7 \times 10^7$ cells lie in the beam.

A7. Single vesicle model and comparison with previously published work

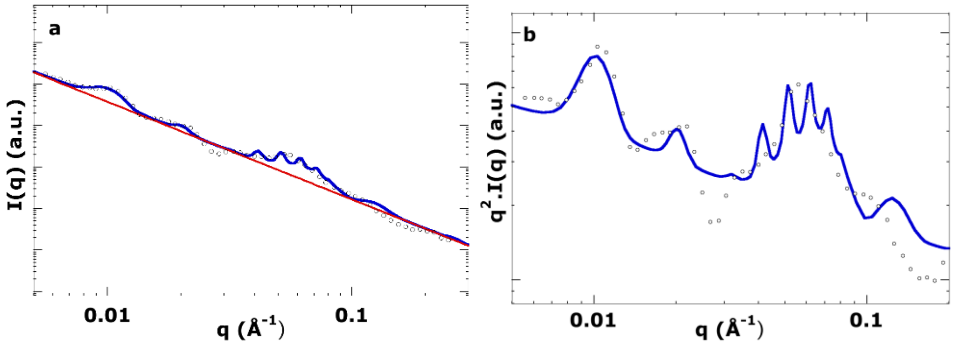

Figure A9a,b Fit to experimental SANS from live *Synechocystis* sp. PCC 6803. Experimental data taken from Jakubauskas *et al.* (2019). The model used for fitting their data here is our own single vesicle model (based on the form factor of equation 2 of the main text), and equations 1 of the main text and equation A1 from this appendix. Our single vesicle model was constructed to be identical to that in Jakubauskas *et al.* (2019). Jakubauskas *et al.*'s experimental data are black circles, the blue line is the fit obtained here. The fit obtained in the present work using the parameters in Table A4 below appears identical to that published by Jakubauskas *et al.* 2019.

Table A4

Fit parameter	*Synechocystis* PC 6803	Fit parameter	*Synechocystis* PC 6803	Fit parameter	*Synechocystis* PC 6803
RD	610 Å	σ_{lumen}	5 Å	n	-2.35
N_{layers}	4	$\Delta\rho_{bilayer}$	-2.00E-06	B	0
η_{Caille}	0.02	$\Delta\rho_{lumen}$	5.00E-07	**Derived parameters**	
$d_{bilayer}$	34 Å	**scale**	1.90E+07	**Stack thickness**	129 Å
d_{lumen}	60.96 Å	C	7.50E-04	**Interstack space**	481 Å

A8. Covariance Analysis for d_{IT} versus $\Delta\rho_{IT}$

To evaluate whether a stress-induced change in the inter-thylakoid gap thickness (d_{IT}) is resolvable within our model, we performed a focused two-dimensional $\chi^2_{min} + 1$ analysis on a 3×3 grid of d_{IT} and its most comparable co-variable, the $\Delta\rho_{IT}$ (inter thylakoid gap SLD contrast). Although we do not claim direct observation of a two-state transition, this analysis demonstrates that the model can distinguish the canonical low- and high-stress gap values at 68% confidence—supporting the conclusion that such a transition would be clearly detectable.

In Appendix A4, we showed how a one-dimensional $\chi^2_{\mathrm{min}} + 1$ analysis gives each parameter's 1σ bound by varying it alone. A fully rigorous profiling—where each of the 14 fit parameters is fixed in turn while re-optimizing all the others—would capture all covariances and yield formally correct joint uncertainties. However, such an approach requires an automated fitting routine, careful specification of physical bounds for every parameter, and hundreds or thousands of repeated fits. Moreover, it can be prone to optimizer traps and may over-inflate errors by chasing irrelevant trade-offs among very soft parameters. As the current study already spans multiple results and technical layers, we isolate the most diagnostically comparable parameter and defer full multi-parameter analysis to a future effort.

Instead, we exploit the fact that only parameters whose fractional uncertainty is comparable to d_{IT}'s (~10%) can meaningfully compensate for its variation (see Table A2 and Figures A1–A4). Much "harder" parameters (<5% error; steep χ^2 curvature) are too tightly constrained to shift appreciably, while much "softer" ones (>50% error) typically correspond to degenerate or weakly sensitive directions in parameter space—adjusting freely but contributing too little to χ^2 to offset changes in d_{IT}. Among all candidates, the IT-gap SLD contrast ($\Delta\rho_{IT}$ ~31% error) most closely matches d_{IT}'s softness, and thus alone suffices to test our stress-detection hypothesis. Once the 3×3 d_{IT} vs. $\Delta\rho_{IT}$ grid showed no additional broadening, further partner tests became unnecessary.

Furthermore, while we did not systematically test all pairwise covariances, prior modelling experience indicates that most parameters influence q-space regions distinct from the mid-q oscillations that constrain d_{IT}. For example, background level, high-q slope, and contrast scaling primarily modulate the intensity baseline or envelope, rather than fringe position. As a result, these parameters would be unlikely to covary meaningfully with d_{IT} within the χ^2 landscape, and were not included in the present covariance scan. $\Delta\rho_{IT}$ is the only parameter

that satisfies both the sensitivity and coupling conditions necessary for meaningful covariance with d_{IT}.

In our model, we test whether a stress-induced expansion of the inter-thylakoid (IT) gap—previously inferred from ultrastructural data by Slavov *et al.* (2016)—would be resolvable within our SANS-based fitting framework. Specifically, we evaluate the detectability of an increase of ~4.6 nm in d_{IT}, consistent with Slavov *et al.*'s interpretation of PSI ingress into the IT space under stress. The physical plausibility of such a mechanism is supported by structural studies. Kitmitto *et al. (*1997) reported that higher plant PSI spans ~8–9 nm perpendicular to the membrane, implying stromal extensions of several nanometers. More recently, Lin *et al.* (2024) resolved the PSI structure of *Symbiodinium*, identifying stromal-facing subunits such as PsaT and extended domains that clearly protrude from the membrane into the stroma. These features are structurally consistent with the scale of membrane separation proposed in our test hypothesis.

We therefore constructed two 3×3 grids, centred respectively on the low-stress best-fit d_{IT} (16.6 Å ± 1.7 Å) and the literature-based high-stress gap (40 Å ± 5 Å), each spanning [–1σ, 0, +1σ] in both d_{IT} and $\Delta\rho_{IT}$. At each of the nine (d_{IT}, $\Delta\rho_{IT}$) combinations, we computed χ^2 against the SANS dataset, setting the threshold $\chi^2_{thr} = \chi^2_{min} + 1$ to define the 68% confidence region in this two-parameter space. We then projected the in-contour points onto the d_{IT} axis to extract the covariance-corrected bounds.

In the low-stress grid, the minimum $\chi^2 = 4.303$ occurs at $d_{IT} = 16.62$ Å and $\Delta\rho_{IT} = 9.24\times10^{-7}$ Å^{-2}. Applying $\chi^2_{thr} = 5.303$ identifies acceptable fits at $d_{IT} = 14.92$, 16.62, and 18.32 Å, reproducing the single-parameter result of 16.62 ± 1.70 Å. This agreement confirms that d_{IT} remains tightly constrained even under two-parameter covariance.

In the high-stress grid, the minimum $\chi^2 = 72.608$ occurs at $d_{IT} = 35.00$ Å and $\Delta\rho_{IT} = 6.34\times10^{-7}$ Å^{-2}—on the edge of the tested domain. As this may not represent a true local minimum, the formal criterion $\chi^2 \leq \chi^2_{min} + 1$ is not strictly applicable. However, this limitation does not affect the outcome: all tested combinations with $d_{IT} = 40$ Å or greater yield χ^2 values more than 30 units above the minimum—far beyond the 68% threshold. Thus, even allowing for covariance with $\Delta\rho_{IT}$ the canonical high-stress gap is clearly excluded. The model is demonstrably capable of resolving a two-state d_{IT} transition, and the high-stress configuration is not supported by the data taken of *ex hospite Symbiodinium* derived from *Aiptasia* at 20°C.

A future global profiling of all fit parameters may further validate this result, but current evidence strongly supports $\Delta\rho_{IT}$ as the only meaningful covariant partner for d_{IT} under realistic compositional and structural constraints.

Table A5. Low-Stress Grid

d_{IT} (Å)	$\Delta\rho_{IT}$ (×10⁻⁷ Å⁻²)	χ^2
**14.92**	6.34	7.216
**14.92**	9.24	4.919
**14.92**	12.14	4.333
**16.62**	6.34	5.084
**16.62**	9.24	4.303
**16.62**	12.14	5.518
**18.32**	6.34	4.655
**18.32**	9.24	5.646
**18.32**	12.14	8.912

Table A6. High-Stress Grid

d_{IT} (Å)	$\Delta\rho_{IT}$ ($\times 10^{-7}$ Å^{-2})	χ^2
**35.00**	6.34	72.608
**35.00**	9.24	97.610
**35.00**	12.14	128.37
**40.00**	6.34	108.32
**40.00**	9.24	141.80
**40.00**	12.14	183.40
**45.00**	6.34	145.72
**45.00**	9.24	188.57
**45.00**	12.14	242.94
